# Development of biodegradable Zn-1X binary alloys with nutrient alloying elements Mg, Ca and Sr

**DOI:** 10.1038/srep10719

**Published:** 2015-05-29

**Authors:** H. F. Li, X. H. Xie, Y. F. Zheng, Y. Cong, F. Y. Zhou, K. J. Qiu, X. Wang, S. H. Chen, L. Huang, L. Tian, L. Qin

**Affiliations:** 1 State Key Laboratory for Turbulence and Complex System and Department of Materials Science and Engineering, College of Engineering, Peking University, Beijing 100871, China; 2 Musculoskeletal Research Laboratory, Department of Orthopaedics & Traumatology, The Chinese University of Hong Kong, Shatin, Hong Kong, China; 3 The Department of Orthopedics, The First Affiliated Hospital of Soochow University, Suzhou 215006, China; 4 The Department of Orthopedics, Zhongda Hospital, the Southeast University, Nanjing, China; 5 College of Physics, Peking University, Beijing 100871, China; 6 Center for Biomedical Materials and Engineering, Harbin Engineering University,Harbin, China

## Abstract

Biodegradable metals have attracted considerable attentions in recent years. Besides the early launched biodegradable Mg and Fe metals, Zn, an essential element with osteogenic potential of human body, is regarded and studied as a new kind of potential biodegradable metal quite recently. Unfortunately, pure Zn is soft, brittle and has low mechanical strength in the practice, which needs further improvement in order to meet the clinical requirements. On the other hand, the widely used industrial Zn-based alloys usually contain biotoxic elements (for instance, ZA series contain toxic Al elements up to 40 wt.%), which subsequently bring up biosafety concerns. In the present work, novel Zn-1X binary alloys, with the addition of nutrition elements Mg, Ca and Sr were designed (cast, rolled and extruded Zn-1Mg, Zn-1Ca and Zn-1Sr). Their microstructure and mechanical property, degradation and *in vitro* and *in vivo* biocompatibility were studied systematically. The results demonstrated that the Zn-1X (Mg, Ca and Sr) alloys have profoundly modified the mechanical properties and biocompatibility of pure Zn. Zn-1X (Mg, Ca and Sr) alloys showed great potential for use in a new generation of biodegradable implants, opening up a new avenue in the area of biodegradable metals.

Biodegradable metals have attracted considerable attention in recent years, with two major alloying systems. The first one is Mg and its alloys^1^,^2^,^3^,^4^ including Mg-Ca^5^, Mg-RE^6^,^7^, Mg-Sr^8^, Mg-Zn^9^,^10^ and Mg-based bulk metallic glasses (BMGs)^11^,^12^. The other alloy system is Fe and its alloys, which started to draw researcher’s attention since more than a decade ago^13^. For Fe-based biodegradable metals, Fe-Mn^14^, Fe-Pd^15^, Fe-W^16^, Fe-CNT^16^ and Fe-C^17^ have been studied and developed. However, both of these biodegradable alloy systems have disadvantages in the clinical applications. For Mg and its alloys, their corrosion rates are generally faster than clinical needs and rapid loss of mechanical strength due to fast corrosion could further lead to failure implantation^18^; on the other hand, due to the too fast corrosion reaction Mg + 2H_2_O → Mg^2+ ^+ 2OH^− ^+ H_2_ of Mg and Mg alloys under physiological conditions, unwanted, possibly harmful hydrogen cavities occur since more hydrogen is produced per time interval than it can be dissolved in the surrounding tissue or diffuse from the implant surface into the extracellular medium^19^, and these gas bubbles impede the good connectivity of osteocytes, interfered with the initial cortical bone healing process, resulting in callus formation and cortical defects, thus has abated the optimistic predictions for its use in osteosynthesis^20^. For Fe and its alloy, on the contrary, their corrosion rates are generally lower than clinical needs and would stay for a long time in human body even after finishing their clinical role^21^.

Patrick K. Bowen *et al*^22^. studied the corrosion rate of pure Zn wire in the abdominal aorta of adult male Sprague–Dawley rats, and their results demonstrated that Zn had better corrosion rate for clinical applications compared with Fe and Mg alloys. Besides the ideal corrosion rate property for biodegradable alloys, the significance of zinc in human nutrition has been proven by various studies. Zinc is an essential element for basic biological function, and it participates in nucleic acid metabolism, signal transduction, apoptosis regulation, and gene expression in addition to interacting with a variety of organic ligands. It is well established that zinc plays an important role in growth and stimulates bone formation, mineralization and plays a role in the preservation of bone mass. Zinc was reported a highly potent and selective inhibitor of osteoclastic bone resorption *in vitro*^23^. Zinc content in bone matrix decreased in conditions like aging and skeletal disease^24^. The number of biological functions, health implications and pharmacological targets granted zinc as “the calcium of the twenty-first century”^25^. However, pure Zn is soft, brittle and has low mechanical strength; it was reported that the tensile strength of the as cast Zn was below 20 MPa^26^, the elongation was only 0.2%^26^, and the Vickers hardness was 37^27^. Thus further studies are needed to achieve the desired mechanical properties. D. Vojtěch *et al*^26^. developed as cast Zn-Mg alloys and studied their mechanical and corrosion behavior, and their results showed that Mg alloying at 1% (wt.%) significantly improved the tensile strength of Zn, however, the elongation of as cast ZnMg alloy was only 2% in spite of a significant improvement for Zn with an initial 0.2% elongation.

Till now, the study of this new type of potential biodegradable material is lacking. Considering the biosafety aspect, elements with potential toxicological problems should be avoided in the initial design stage for biodegradable metals. The previous widely used commercial Zn alloys are developed and designed for industrial applications. Some of the alloying elements existing in the commercial Zn alloys usually have biosafety concern. For instance, ZA series contain toxic Al elements up to 40% (wt.%). It is well known that Al is harmful to neurons, bone and osteoblasts^28^, associated with dementia^29^,^30^ and Alzheimer’s disease^31^. Consequently, Al is unsuitable alloying elements for biomedical materials. This resulted in development of novel biodegradable Zn-based alloys without Al or other harmful elements. In order to secure the biosafety of biomedical Zn alloys and consider the skeletal applications, we chose the nutrient elements (Mg, Ca and Sr) that associated closely with bone tissue as alloying element for Zn in the present study. It is well known that Mg, Ca and Sr are in the same group (IIA) in the periodic table, and are the important elements of bone matrix. Mg regulates active calcium transport and thus increases bone mineral density and prevents bone fracture^32^. Calcium intake is one of the important modifiable environmental factors for the normal development of the skeleton during growth and the maintenance of bone mass in later life^33^. The distribution of Sr is similar to Ca, with 99% of the element being stored in bone^34^. Strontium can inhibit bone resorption and stimulate bone formation in both rodents and osteoporotic patients^35^.

In the present study, we choose the three important essential nutrient elements Mg, Ca and Sr, which are well known playing an irreplaceable role in bone formation, and determining bone mineral density and bone strength^33^,^35^,^36^, as alloying elements to fabricate the Zn-1X binary alloy casting ingots. Besides adding alloying elements to form alloys, another important factor that would significantly influence the properties of materials is the history they went through, i.e. the preparation process. It has been widely reported that the alloys treated by rolling and extrusion are generally gain enhanced mechanical and corrosion behavior. Considering that, different hot working conditions (rolling and extrusion) were employed on the Zn-1X (Mg, Ca and Sr) alloys in the present study in order to further improve their mechanical performance. The microstructure, mechanical properties, corrosion behavior, hemocompatibility, *in vitro* cytocompatibility and *in vivo* biocompatibility were studied systematically to investigate their feasibility as bioabsorbable implants for orthopedic applications.

## Results

### Composition and microstructural characterization

**Figure 1:**
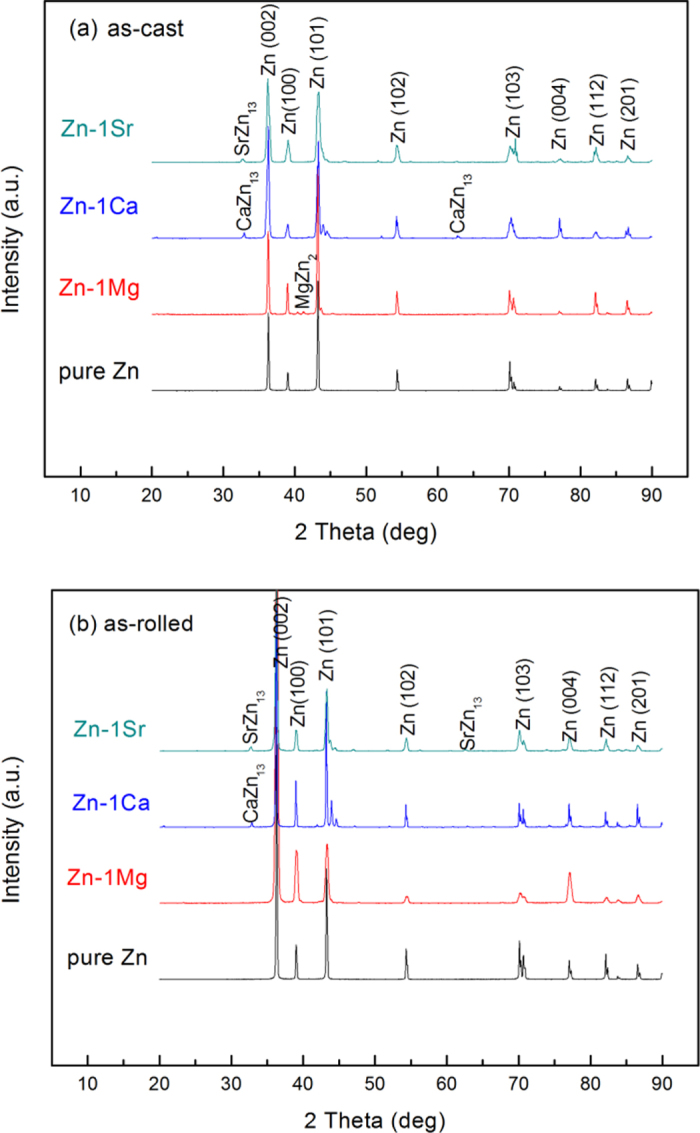
XRD patterns of pure Zn and Zn-1X (Mg, Ca, Sr) binary alloys: (**a**) as cast; (**b**) as rolled.

The chemical compositions of the Zn-1X binary (Mg, Ca, Sr) alloys were analyzed by ICP-AES. The content of alloying elements Mg, Ca and Sr in corresponding Zn-1X binary alloys are 1.15 ± 0.03 wt.% (Zn-1Mg), 1.05 ± 0.02 wt.% (Zn-1Ca) and 1.11  ± 0.02 wt.% (Zn-1Sr), respectively. Figure 1 shows the XRD patterns of as-cast (a) and as-rolled (b) pure Zn and Zn-1X (Mg, Ca and Sr) binary alloys. It can be found that pure Zn and Zn-1X (Mg, Ca, Sr) binary alloys are mainly composed of Zn with a hexagonal close packed (hcp) crystal structure, besides, for the binary alloys, MgZn_2_, CaZn_13_ and SrZn_13_ were also detected.

### Mechanical properties

**Figure 2:**
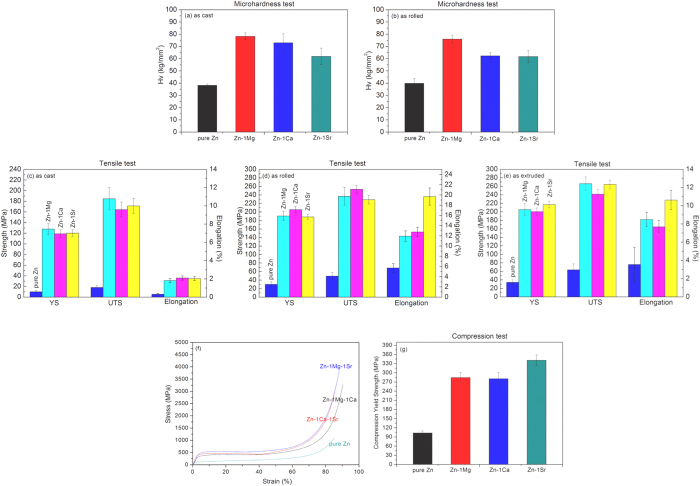
Mechanical properties of pure Zn and Zn-1X (Mg, Ca, Sr) binary alloys: (**a**) and (**b**) Microhardness; (**c**), (**d**) and (**e**) Tensile properties; (**f**) and (**g**) Compressive properties of as extruded pure Zn and Zn-1X (Mg, Ca, Sr) binary alloys (**f**) Stress-Strain curves; (**g**) Compressive Yield Strength (YS).

Figure 2a,b shows the microhardness of as-cast (a) and as-rolled (b) pure Zn and Zn-1X (Mg, Ca, Sr) binary alloys. It is obvious that the as-cast pure Zn has quite low hardness, only (38.24 ± 1.06) Hv, but after alloying with Mg, Ca and Sr elements, the microhardness is enhanced significantly to (78.26 ± 2.84) Hv (as-cast Zn-1Mg), (73.00 ± 7.43) Hv (as-cast Zn-1Ca) and (61.88 ± 6.72) Hv (as-cast Zn-1Sr), respectively. The microhardness of the as-rolled Zn-1X alloys has similar trend with that of the as-cast alloys.

Figure 2c,d,e shows the tensile properties of as-cast (c), as-rolled (d) and as-extruded (e) experimental pure Zn and Zn-1X (Mg, Ca, Sr) binary alloys. It is obvious that the yield strength (YS), ultimate tensile strength (UTS) and elongation of as-cast pure Zn are low, with only (10.14 ± 2.32) MPa, (18.25 ± 2.99) MPa, and (0.32 ± 0.08)%, respectively. Adding the alloying elements Mg, Ca and Sr, the YS, UTS and elongation of as-cast Zn-1X binary alloys are significantly improved to (127.98 ± 10.72) MPa, (184.84 ± 20.91) MPa and (1.82 ± 0.23) % (Zn-1Mg); (119.12 ± 7.01) MPa, (164.57 ± 13.92) MPa and (2.10 ± 0.23)% (Zn-1Ca); (120.21 ± 6.08) MPa, (171.40 ± 14.13) MPa and (2.03 ± 0.22)% (Zn-1Sr). After hot rolling, the YS, UTS and elongation of Zn-1X binary alloys are further improved up to (253.30 ± 9.36) MPa (as-rolled Zn-1Mg) and (19.69 ± 1.68) % (as-rolled Zn-1Sr). The hot extrusion process also improved the tensile properties of the Zn-1X alloys, similar to that of hot rolling process.

Figure 2f,g shows the compressive properties of pure Zn and Zn-1X (Mg, Ca, Sr) binary alloys. From the compression stress-strain curves shown in Fig. 2f, it can be noted that the Zn-1X binary alloys exhibit unique superplastic characteristic under compression, which was explained by the formation of compressive twin crystal^37^. Because of the compression superplastic characteristic of the Zn-1X binary alloys, we are unable to obtain the ultimate compressive strength and only get the yield strength under compression. Figure 2g shows the compressive yield strength data of the pure Zn and Zn-1X binary alloys. It is obvious that the compressive yield strength has been improved from (102.92 ± 6.73) MPa for pure Zn to (284.50 ± 16.90) MPa (Zn-1Mg), (280.70 ± 20.72) MPa (Zn-1Ca), (340.98 ± 17.70) MPa (Zn-1Sr), suggesting the effectiveness of alloying (Mg, Ca and Sr elements) on the improvement of its mechanical property.

Mechanical property is a very important factor for biodegradable metals. The implants, such as bone plates and vascular stents, are used to substitute for the human tissue, which means that their mechanical performance should match the mechanical performance or environment of the local tissues or organs.

The as-cast pure Zn has quite poor mechanical properties, including hardness, tensile and compression strength, thus it could not meet the biomedical applications. In order to enhance the mechanical properties of pure Zn, the two common used methods in metallic materials processing - adding alloying elements and hot-working deformation are tested in the present work. As shown in Fig. 2, these two methods both perform effectively well.

### Corrosion behavior

**Figure 3:**
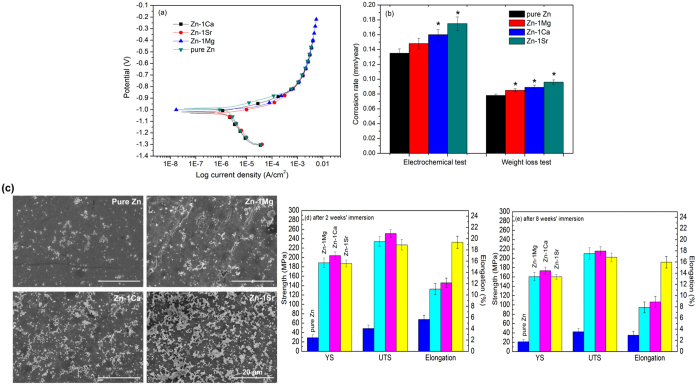
Corrosion behavior of as rolled pure Zn and Zn-1X (Mg, Ca, Sr) binary alloys: (**a**) Electrochemical polarization curves; (**b**) Corrosion rate generated by electrochemical tests and weight loss tests (* means p < 0.05 compared with pure Zn group); (**c**) Surface morphology of as rolled pure Zn and Zn-1X alloys after immersed in Hank’s solution for 2 weeks; (**d**) and (**e**) Tensile tests of as rolled pure Zn and Zn-1X (Mg, Ca, Sr) after immersion in Hank’s solution for 2 weeks (**d**) and 8 weeks (**e**).

Figure 3a shows the electrochemical polarization curves of as-rolled experimental materials. From the electrochemical polarization curves, we can get the corrosion potentials (E_*corr*_) and corrosion current density *(i*_*corr*_). The corrosion potentials (E_*corr*_) of Zn-1X binary alloys are −999 mV (Zn-1Mg), −1019 mV (Zn-1Ca) and −1031 mV (Zn-1Sr) respectively, lower than that of pure Zn (−988 mV), suggesting the Zn-1X binary alloys are more likely to develop corrosion than pure Zn. Correspondingly, via linear fit and Tafel extrapolation to the cathodic and anodic part of the polarization curves, we got the corrosion current density *(i*_*corr*_) of Zn-1X binary alloys as 9.94 μA/cm^2^(Zn-1Mg),10.75 μA/cm^2^ (Zn-1Ca), 11.76 μA/cm^2^ (Zn-1Sr), higher than that of pure Zn (9.07 μA/cm^2^). Figure 3b shows the corrosion rate data calculated from the electrochemical measurement and weight loss by immersion testing. The corrosion rate of Zn-1X alloys are significantly higher than that of pure Zn (*p < 0.05) and the sequence is Zn < Zn-1Mg < Zn-1Ca < Zn-1Sr. Figure 3c shows the surface morphology of pure Zn and Zn-1X alloys after immersing in Hank’s solution for 2 weeks, where the pure Zn and Zn-1X alloys remain flat surface after 2 weeks’ immersion in Hank’s solution simulated body fluid. After 8 weeks’ immersion, the surfaces were covered with corrosion products and XPS analysis indicated the HA deposited on the top surface. (presented in the Supplementary Information as S1).

Figure 3d,e shows the tensile properties of as rolled pure Zn and Zn-1X alloys after 2 weeks’ (d) and 8 weeks’ (e) immersion in Hank’s simulated body fluid. The tensile strength of Zn-1X alloys still remains a high level with a slight decline after 8 week’s degradation in Hank’s solution simulated body fluid. As orthopedic applications such as for fracture repair, it usually needs 3–4 months to keep the mechanical properties, e.g. in fracture repair starting from fracture callus formation to mineralization and remodeling, i.e., the bone implant should maintain its mechanical property at the first few months after implantation to avoid the occurrence of second fracture^38^. The main problem of Mg alloys as bone implant is the fast loss of strength during the early degradation stage. It has reported that the bending strength of Mg-Zn alloy decreased fast to half of the original strength at the initial corrosion stage^18^. The loss of mechanical integrity during degradation may inhibit the clinical realization of Mg-based alloys. Different from the Mg-based alloys, the present Zn-1X alloys can remain the mechanical strength during the early implantation time. This strength surviving feature may provide additional advantage of the biodegradable Zn-1X alloys for bone implantation.

### *In vitro* biological compatibility

**Figure 4:**
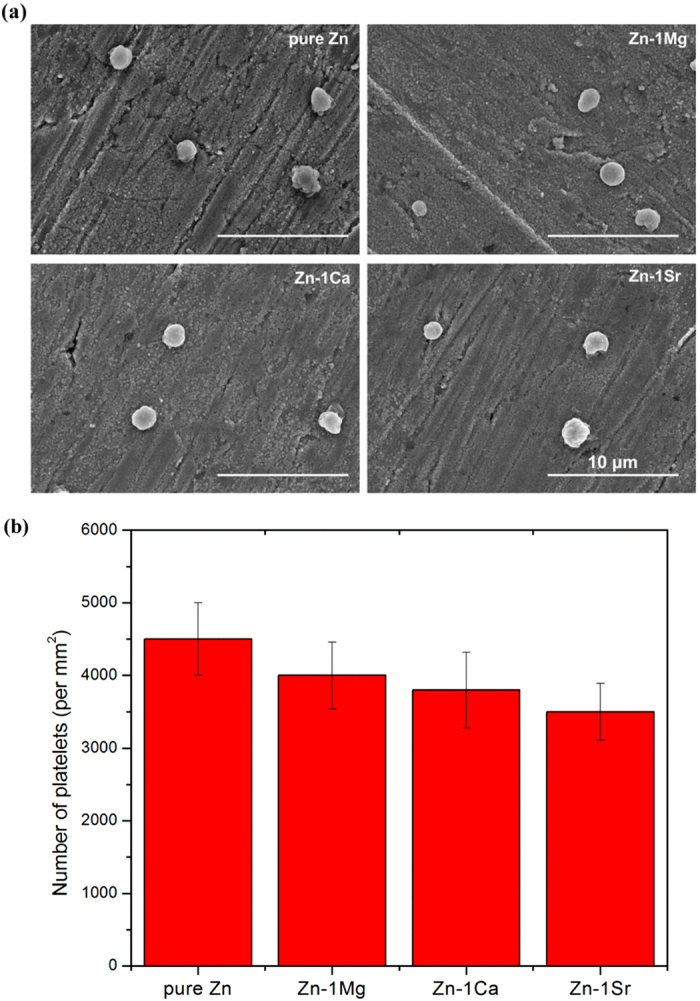
(**a**) Morphologies and (**b**) Number of adhered platelets on pure Zn and Zn-1X alloys.

**Figure 5:**
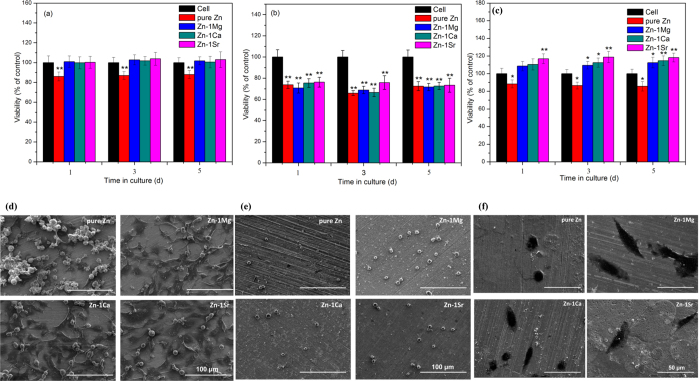
(**a**) ECV304, (**b**)VSMC, (**c**) MG63 cell viability cultured in cell culture medium, pure Zn and Zn-1X alloy extracts (* means p < 0.05, ** means p < 0.01 compared with pure Zn group); (**d**) ECV304, (**e**)VSMC, (**f**) MG63 cell morphologies cultured directly on pure Zn and Zn-1X alloy surfaces for 1d.

Our study shows that the hemolysis rates of Zn-1X alloys are quite low (<0.2%) (Supplementary Information S2), which are far below the safe value of 5%, suggesting that the experimental Zn-1X alloys would not lead to severe hemolysis according to ISO 10993–4:2002. Figure 4a shows the morphologies of adhered platelets on pure Zn and Zn-1X alloy plates. The results demonstrate that the platelets present round shape with no pseudopodia spreading. These normal morphologies of adhesive platelets on Zn-1X alloys indicate their excellent *in vivo* anti-platelets adhesion property and antithrombotic properties^39^. Figure 4b enumerates the adhered platelets on top of pure Zn and Zn-1X alloy plates, which are about 3500 ~ 4500/mm^2^. It is obvious that the addition of alloying elements into Zn can reduce its adhesive platelets number, but there is no statistical significance between the Zn-1X alloy and pure Zn (p > 0.05). Figure 5a shows the viability of ECV304 cells and Fig. 5b shows the viability of VSMC cells cultured in pure Zn and Zn-1X alloy extraction medium for 1, 3 and 5 days. For ECV304 cell, the Zn-1X alloy groups showed significantly high cell viabilities in comparison to Pure Zn group, whereas for VSMC cell, the alloying elements don’t have obvious promotion effect. Figure 5c shows the change of viability of MG63 cell with time when cultured with various experimental material extracts. It can be seen that the extracts of pure Zn can lead to significantly reduced cell viability (*p < 0.05) in comparison with negative controls, however, adding the alloying elements Mg, Ca and Sr into Zn can significantly increase the viability of MG63 and can promote the MG63 cell proliferation compared with the pure Zn and negative control groups. Moreover, the alloying element Sr can promote the MG63 cell proliferation at the best effect, Ca lies in the second and followed by the Mg alloying elements. Figure 5d–f shows the SEM observation of the ECV304 (Fig. 5d), VSMC (Fig. 5e) and MG63 (Fig. 5f) cell morphologies cultured directly on the pure Zn and Zn-1X alloy plates for 1 d. It is obvious that the cells exhibit an unhealthy morphology cultured on pure Zn, most of them exhibited round and dead, only few cells showed healthy polygonal or spindle shaped. However, the ECV304 and MG63 cell morphologies are healthy on the Zn-1X alloy surfaces, most of them exhibited polygonal or spindle shape and well spread and proliferated, and they also exhibited affluent pseudopods and secreted extracellular matrix. Unlike the MG63 cells and ECV304 cells, the VSMC cells don’t adhere, spread and proliferate on all experimental group surfaces. The VSMC cells showed an unhealthy round and dead morphology on pure Zn and Zn-1X alloy surfaces. It indicated that the addition of Mg, Ca and Sr alloying elements to Zn doesn’t improve the cell adhesion, viability and proliferation for VSMC.

### *In vivo* biological compatibility

**Figure 6:**
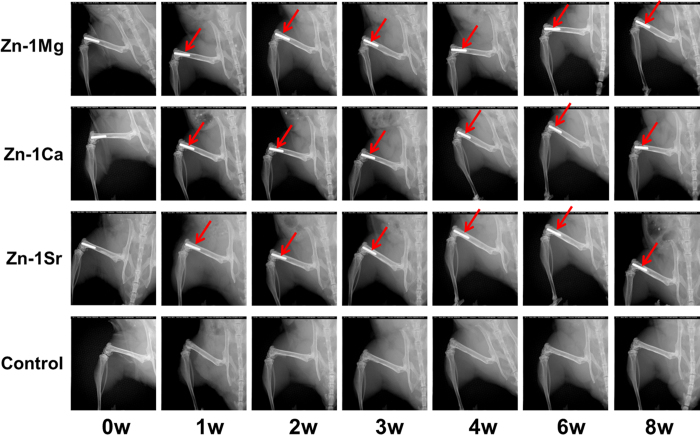
Radiographs of mouse femora with implantation of Zn-1X alloy pins at week 0, 1, 2, 3, 4, 6, and 8.

**Figure 7:**
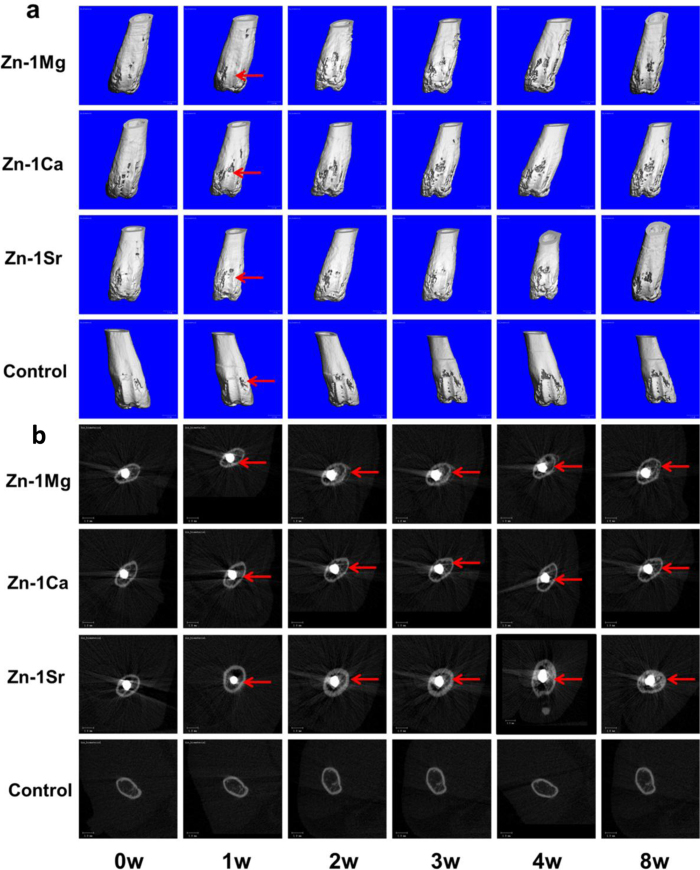
Micro-CT 3D (**a**) and 2D (**b**) images of Zn-1X pin groups and the sham control group at week 0, 1, 2, 3, 4 and 8.

**Figure 8:**
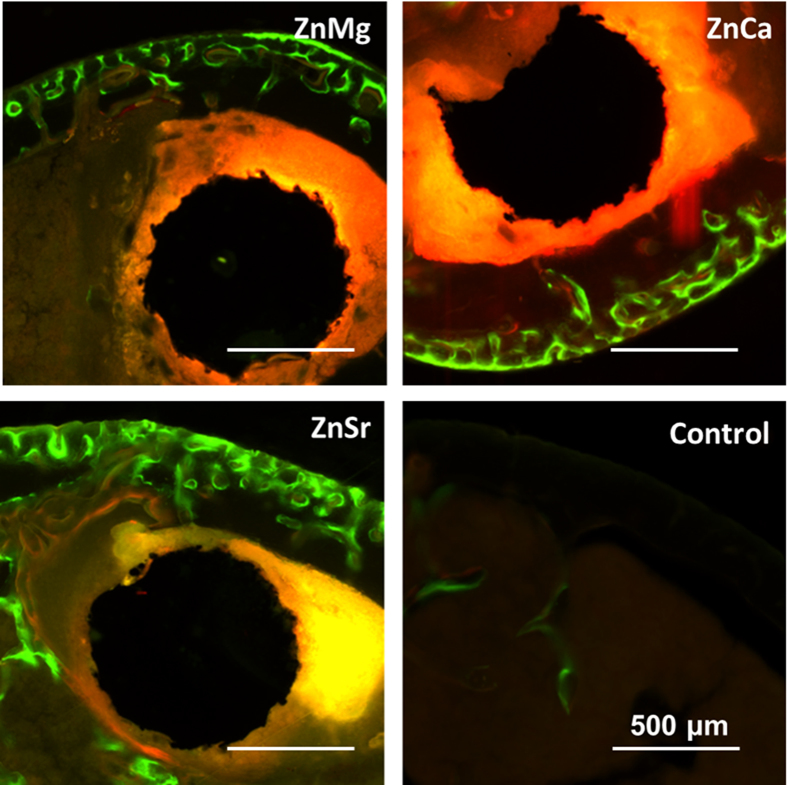
Representative histology of the cross-sections of mouse distal femoral shaft from Zn-1Mg, Zn-1Ca, Zn-1Sr implanted pins groups and the sham control group observed under fluorescent microscopy at week 8.

Figure 6 shows the radiographs of mouse femora with implantation of Zn-1X alloy pins evaluated at 0, 1, 2, 3, 4, 6 and 8 weeks. There is no inflammation observed around the implantation site and there is no mouse died after operation. Zn-1X alloy pins degraded slowly during the whole experiment period. If a material degraded fast *in vivo*, the large amount of hydrogen gas generated would not be absorbed or diffusion completely in a short period as confirmed by X-ray radiographs with gas shadow. In the present study, due to the relatively slow degradation rate *in vivo*, gas shadow, which was caused by generation of hydrogen gas bubbles during degradation, was not observed around the pins. The profiles of Zn-1X pins were distinct indicating their excellent radiopacity, which is in fact essential for interventional and minimally invasive surgery. After a short period (after 1 week), the periosteal reaction and reactive hyperplasia of bone (red arrows) were observed around the cortical bone of distal femur in Zn-1X pins groups and showed the circumferential osteogenesis, thus the cortical bone around the pins became thicker than that in the sham control group, indicating that the Zn-1X pins could promote new bone formation when compared to that in the sham control group. Figure 7a shows the Micro-CT 3D images of Zn-1X pin groups and that of the sham control group. It is obvious that after 1 week, the continuous changes of bone (new bone formation and remodeling) at the surface of the distal femora (red arrows) are found in Zn-1Mg, Zn-1Ca, Zn-1Sr pin groups. No obvious change is observed in the sham control group. The bone volume in Zn-1Mg, Zn-1Ca and Zn-1Sr pin groups is higher than that in the sham control group after week 1, and the bone volume in Zn-1Sr group is the highest. Figure 7b shows the 2 D cross-section images from Micro-CT of distal femora with Zn-1X pins and without metal alloy pins (sham control group) at week 0, 1, 2, 3, 4 and 8 after surgical implantation. The change of bone matrix with new bone formation (red arrows) is found at week 1 after operation. The micro-CT results show that the integrity of Zn-1Mg, Zn-1Ca, Zn-1Sr alloy pins are maintained from 0 week to 8 weeks. According to the Micro-CT analysis, we can get the *in vivo* corrosion rates of the Zn-1X alloys, which are 0.17mm /year, 0.19 mm/year and 0.22 mm/year for Zn-1Mg, Zn-1Ca and Zn-1Sr pins respectively. Figure 8 shows the representative histological cross-sectional images under fluorescent microscopy. The green fluorescence indicating the new bone formation. It is obvious that the cortical bone close to the Zn-1X alloy pin was thicker than that in the sham control group, and more new bone formed at periosteum in Zn-1Mg, Zn-1Ca and Zn-1Sr pin groups, especially in Zn-1Sr alloy pin group when compared to that in the sham control group. The new bone thickness of the sham control group is only (25.8 ± 10.23) μm. On the contrary, the new bone thickness of Zn-1Mg, Zn-1Ca and Zn-1Sr pin groups are significantly larger than the sham control group (**p < 0.01), (188.26 ± 20.10) μm, (233.00 ± 22.00) μm and (364.20 ± 26.00) μm, respectively. (Supplementary Information S3).

## Discussion

Biodegradable metals are breaking the current paradigm in biomaterial science to develop only corrosion resistant metals. In particular, metals which consist of trace elements existing in the human body are promising candidates for this approach^19^. Pure Zn has been recently studied as new kind of biodegradable material. However, pure Zn is soft, brittle and has low mechanical strength. Thus further studies are needed to achieve the desired mechanical properties. Alloying and modifying the processing history are the two mostly used methods in metallic materials for property improvement. Considering the biosafety aspect, elements with potential toxicological problems should be avoided in the initial design stage for biodegradable metals. It is well known that Mg, Ca and Sr are in the same group (IIA) in the periodic table, and are the important elements of bone matrix. In order to secure the biosafety of biomedical Zn alloys and consider the skeletal applications, we chose the nutrient elements (Mg, Ca and Sr) that associated closely with bone tissue as alloying element for Zn in the present study. Besides the enhanced mechanical properties of the Zn-1X binary alloys, both the *in vitro* electrochemical data and *in vivo* data showed that the corrosion rate of the Zn-1X binary alloys is a good fit for orthopedic application, as it guarantees the sufficient mechanical support during the tissue repair process.

In summary, we developed Zn-X binary alloys for use as biodegradable materials within bone. It has been demonstrated that the Zn-1X alloys have greatly enhanced the mechanical properties, corrosion behavior and biocompatibility of pure Zn by adding the alloying elements of Mg, Ca and Sr. Zn-1X alloys showed great potential for use in a new generation of biodegradable implants, opening up a new avenue in the area of biodegradable metals.

## Methods

### Materials preparation

Zn-1X (Zn-1Mg, Zn-1Ca and Zn-1Sr, wt.%) binary alloy ingots were prepared from pure Zn (99.99%) ingots, pure Mg (99.98%) ingots, pure Ca (99.9%) ingots and pure Sr (99.9%) ingots in a high purity graphite crucible under the protection of a mixed gas atmosphere of SF_6_ (1 vol.%) and CO_2_ (balance). After being held at 630 °C for 30 min the melt was poured into a steel mold preheated to 150 °C. In order to increase the mechanical properties, the as-cast pure Zn and Zn-1X binary alloy ingots were further hot-rolled and hot-extruded. For the as-rolled samples, the as-cast pure Zn and Zn-1X binary alloy ingots were cut into 8 mm thick plates. These plates were pre-heated to 250 °C for 3 h, followed by rolling down to 1.5 mm thick sheets. The reduction in thickness of the plates was 0.2 mm in a single pass and the plates were reheated to 250 °C for 10 min between each pass. For the as-extruded samples, the as-cast pure Zn and Zn-1X binary alloy ingots were cut into φ28 mm cylinder. These cylinders were pre-heated to 210 °C for 3 h, followed by extrusion down to φ10 mm cylinder. Disk samples (10 × 10 × 1.5 mm^3^) for microstructure characterization, corrosion experiments, hemolysis, platelet adhesion, cell experiments were cut from the rolled sheets parallel to the rolling direction. Cylindrical rods with a diameter of 0.7 mm and a length of 5 mm were machined from the as-rolled plates as intramedullary nails for implantation into mice. All samples were grounded with SiC paper up to 2000 grit, followed by ultrasonic cleaning in acetone, absolute ethanol and distilled water for 15 min each. For the cytocompatibility tests, the samples were sterilized by ultraviolet-radiation for at least 2 h for one side and then turn over the samples for another 2 h of ultraviolet radiation sterilization.

### Microstructural characterization and composition analyses

X-ray diffractometer (XRD, Rigaku DMAX 2400, Japan) using Cu K_α_ radiation at a scan rate of 4°/min operated at 40 kV and 100 mA at room temperature was employed for the identification of the microstructure of pure Zn and Zn-1X binary alloy samples. The chemical compositions of the resulting alloy ingots were analyzed by inductively coupled plasma atomic emission spectrometry (ICP-AES) (Profile, Leeman Labs, Hudson, NH, USA)^8^.

### Mechanical tests

The tensile and uniaxial compression testing samples were prepared according to ASTM standards from the rolled sheets and the extruded cylinders. The tensile and uniaxial compression tests were performed at room temperature in accordance with ASTM-E8M-09 and ASTM E9-89a (2000) standards respectively using a universal material test machine (Instron 5969, USA). The tensile properties of the Zn-1X alloys after immersion in Hank’s solution simulated body fluid were also measured using a universal material test machine (Instron 5969, USA). Five parallel specimens were taken for each group. Vickers hardness was determined using a hardness tester (HMV-2T, Shimadzu corporation, Japan), with an applied 100 g load and a loading time of 15 s. Six indentations were made for each sample and the diagonal lengths of the indentations were measured using a calibrated micrometer attached to the eyepiece of the microscope. The Vickers hardness number *H*_V_ is computed using the formula of *H*_V_ = 1.8544*P*/*d*^2^, where *H*v is the Vickers hardness number in kg/mm^2^, *P* is the indenter load in kg and *d* is the diagonal length of the indentation in mm^40^,^41^.

### Immersion tests

Immersion tests were carried out in Hank’s solution simulated body fluid (NaCl 8.0 g, CaCl_2_ 0.14 g, KCl 0.4 g, NaHCO_3_ 0.35 g, glucose 1.0 g, MgCl_2_·6H_2_O 0.1 g, Na_2_HPO_4_·2H_2_O 0.06 g, KH_2_PO_4_ 0.06 g, MgSO_4_·7H_2_O 0.06 g dissolved in 1 L deionized water)^42^ at 37 ℃ according to ASTM-G31-72^43^. The pH value of the solution was recorded during the immersion tests (pH meter, Lei-ci PHS-3C). After 2 weeks’ and 8 weeks’ immersion, samples were removed from Hank’s solution, gently rinsed with distilled water and dried in air. Changes in surface morphology and composition after corrosion were characterized by scanning electron microscopy (SEM, Hitachi S-4800, Japan). The weight of the samples after the corrosion test was measured on an electronic balance (Mettler Toledo AL204) with a measuring sensitivity of 0.1 mg after removal of the corrosion products. An average of five measurements were taken for each group. The *in vitro* corrosion rate was calculated according to the equation: C = ∆m/ρAt, where C is the corrosion rate in mm/year, ∆m is the weight loss, ρ is the density of the material, A is the initial implant surface area and t is the implantation time.

### Electrochemical tests

The electrochemical tests were conducted with an electrochemical workstation (CHI 604D) at the temperature of 37 °C in Hank’s solution. Before electrochemical tests, the surface of the samples were polished. A three-electrode cell was used for electrochemical measurements. The auxiliary electrode was platinum and the reference electrode was a saturated calomel electrode (SCE). The open circuit potentials (OCP) were monitored for 7200 s. The potentiodynamic polarization tests were carried out at a scanning rate of 1 mV/s and the initial potential was about 300 mV below the corrosion potential (E_corr_) after OCP measurements. The measurement area was 0.385 cm^2^ (Φ7 mm). Three measurements were taken for each sample group. Tafel regions of the polarization curve were analyzed to get the corrosion current density (*i*_*corr*_) of pure Zn and Zn-1X alloys (by linear fit and Tafel extrapolation to the cathodic and anodic part of the polarization curves). The *in vitro* corrosion rate calculated by electrochemical measurement was got by converting the *i*_*corr*_ into the corrosion rate based on Faraday’s Laws: C = M *i*_*corr*_/nFρ, where C is the corrosion rate in mm/year, M is the Molar mass, n is the number of electrons involved in the corrosion reaction, F is the Faraday’s constant, ρ is the density of the material.

### Hemocompatibility evaluation

Healthy human blood from a volunteer was applied to conduct hemolysis and platelet adhesion tests. Informed consent was obtained from all subjects. For hemolysis tests, healthy human blood containing sodium citrate (3.8 wt.%) in the ratio of 9:1 was taken and diluted with normal saline (4:5 ratio by volume). Samples were dipped in a standard tube containing 10 ml of normal saline that were previously incubated at 37 °C for 30 min. Then 0.2 ml of diluted blood was added to this standard tube and the mixtures were incubated for 60 min at 37 °C. Similarly, normal saline solution was used as a negative control and deionized water as a positive control. After this period, all the tubes were centrifuged for 5 min at 3000 rpm and the supernatant was carefully removed and transferred to the cuvette for spectroscopic analysis at 545 nm using microplate reader (Bio-RAD680). For platelet adhesion tests, platelet-rich plasma (PRP) was prepared by centrifuging the whole blood for 10 min at a rate of 1000 rpm/min. The PRP was overlaid atop the experimental alloys plates and incubated at 37 °C for 1 h. The samples were rinsed with PBS to remove the nonadherent platelets. The adhered platelets were fixed in 2.5% glutaraldehyde solutions for 1 h at room temperature followed by dehydration in a gradient ethanol/distilled water mixture (50%, 60%, 70%, 80%, 90% and 100%) for 10 min each and dried in hexamethyldisilazane (HMDS) solution. The surfaces of platelet attached experimental alloy plates were observed by SEM (S-4800, Hitachi, Japan). Different fields were randomly counted and values were expressed as the average number of adhered platelets per mm^2^ of surface.

### Cytocompatibility evaluation

Human osteosarcoma MG63 cells, human umbilical vein endothelial cells (ECV304) and rodent vascular smooth muscle cells (VSMC) were adopted to evaluate the cytocompatibility of pure Zn and Zn-1X(Mg, Ca, Sr) binary alloys. Informed consent was obtained from all subjects. The cell culture procedure can be found in reference 21. The cytocompatibility evaluation was conducted by both indirect and direct contact methods. For the indirect contact tests, the cells were cultured in the extracts of Zn alloys. The extracts were prepared using serum free cell culture medium as the extraction medium with the surface area of extraction medium ratio 1.25 ml·cm^−2^ in a humidified atmosphere with 5% CO_2_ at 37 °C for 72 h. The cell viability and proliferation were measured with a Cell Counting KIT-8 (CCK-8, Dojindo, Japan). In order to avoid ion and serum influence, before adding CCK-8 solution, the old cell culture media were replaced by fresh cell culture media without serum. For the direct contact tests, the cells were seeded and cultured directly on the surface of the Zn alloys. The cell morphology were observed by SEM (Hitachi S-4800, Japan) after being fixed in 2.5% glutaraldehyde solutions for 1 h at room temperature and dehydration in a gradient ethanol/distilled water mixture (50%, 60%, 70%, 80%, 90% and 100% ethanol).

### Animal experiment

3-month old C57BL/6 mice were used to examine the *in vivo* degradation properties of the Zn-1X(Mg, Ca and Sr) implants. In brief, the mice were anesthetized using Ketamine (75 mg/kg) and Xylazine (10 mg/kg). A tunnel with 0.7 mm in diameter and 5 mm in length was created along into the medullary cavity along the axis of femoral shaft from distal femur. The sterilized alloy implants with 0.7 mm in diameter and 5 mm in length were implanted into the bone tunnel. After operation, the mice were housed in an environmentally controlled animal care laboratory after surgery. Animal experimental protocol was approved by the Animal Ethics Committee of the Chinese University of Hong Kong (No. 10/049/MIS). The methods were carried out in accordance with the approved guidelines. The *in vivo* analysis include (1) X-ray observation: Every week post-operation, the sequential radiographs of the distal femur were taken (30 kV, 3 s) for general inspection; (2) An *in vivo* micro-computerized tomography (micro-CT) (viva CT40, Scanco Medical AG, Brüttisellen, Switzerland) with a voxel size of 20 μm was used to scan the distal femur of mice at week0, 1, 2, 3, 4 and 8 weeks after operation. The change of Zn-1X implants in bone tunnel was measured using our published protocol^44^. The two-dimensional (2D) images were acquired directly from the scanning and the three-dimensional (3D) structure was reconstructed by the volume of interest (VOI) where an optimized threshold was used to isolate the bone and materials from the background. The density changes of Zn-1X implants and the changes of the cortical bone around the Zn-1X implants were measured on the digitally extracted tissue; (3) histological examination: Fluorescent staining agents calcein green (5mg/kg) and xylenol orange (90 mg/kg) were injected subcutaneously at 14 and 4 days respectively before sacrifice to label the newly formed bone around the metal alloy using our published protocol^45^. Eight weeks after operation, the femora of mouse were harvested and fixed in 10% buffered formalin. After gradient dehydration, the harvested femurs were embedded in methylmethacrylate resin. The resin blocks were cut into 120 μm undecalcified sections perpendicular to the long axis of femoral shaft using a diamond saw (Leica SM2500E). The sections were grounded and polished to a thickness of 70 μm, followed by observation under fluorescence microscopy. After that, the sections were stained with van Gieson stain and then observed under light microscopy and scanning electron miscroscopy (SEM S-4800, Hitachi, Japan) equipped with energy-disperse spectrometer (EDS) attachment. The *in vivo* corrosion rate was calculated according to the equation: C = (V0-Vt)/At. Where C is the corrosion rate, V0 is the alloy volume measured by micro-CT on week 0, Vt is the alloy volume measured by micro-CT at different implantation intervals, A is the initial implant surface area and t is the implantation time based on our established protocol.

### Statistical analyses

Statistical analyses were conducted with SPSS 17.0. Differences between groups were analyzed using one-way ANOVA followed by Turkey test.

## Additional Information

**How to cite this article**: Li, H. F. *et al*. Development of biodegradable Zn-1X binary alloys with nutrient alloying elements Mg, Ca and Sr. *Sci. Rep*. **5**, 10719; doi: 10.1038/srep10719 (2015).

## Supplementary Material

Supplementary Information
