# Supplementary material for: Development of biodegradable Zn-1X binary alloys with nutrient alloying elements Mg, Ca and Sr
Source: Sci Rep. 2015 May 29;5:10719. doi: 10.1038/srep10719 (PMC4448657; doi:10.1038/srep10719)
Supplement: Supplementary Information [file srep10719-s1.doc]

**Supplementary Information**

**Development of biodegradable Zn-1X binary alloys**

**with nutrient alloying elements Mg, Ca and Sr**

H. F. Li, X. H. Xie, Y. F. Zheng, Y. Cong, F. Y. Zhou, K. J. Qiu, X. Wang, S. H. Chen, L. Huang, L. Tian, L. Qin


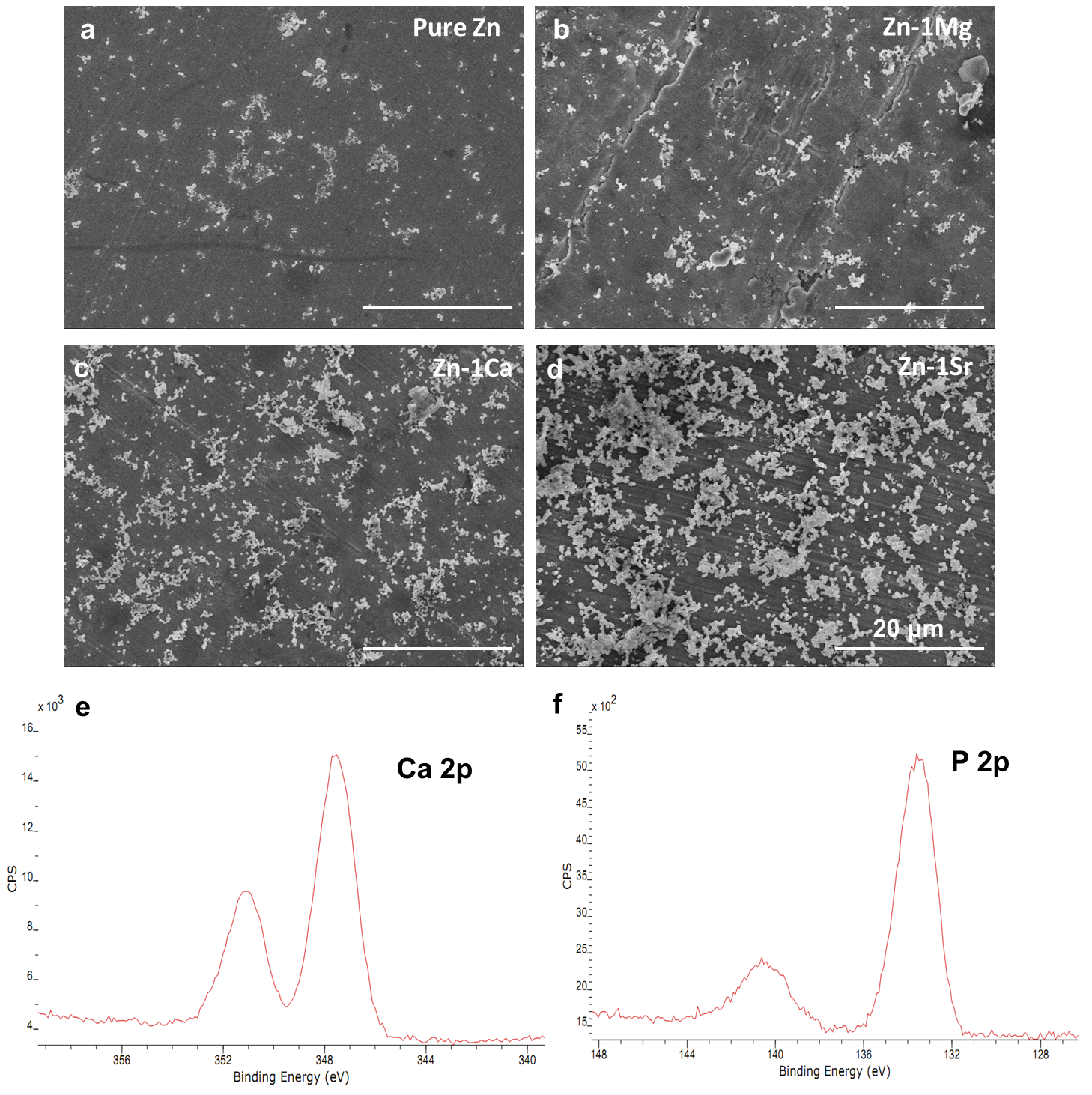

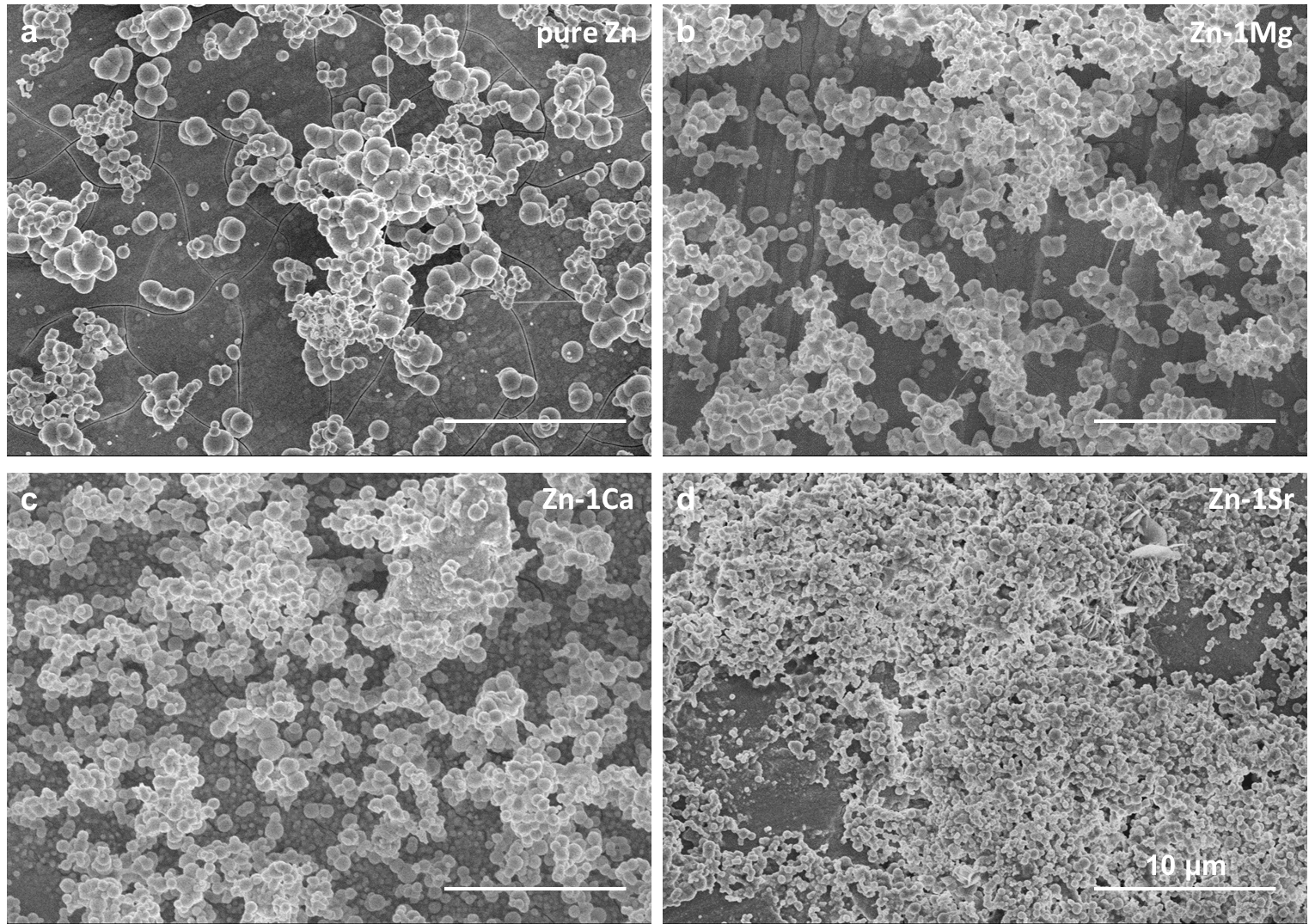


S1 (a-d) Surface morphology of Zn alloys after immersion in Hanks’ solution for 8 weeks, (e-f) XPS analysis of the surface deposition.


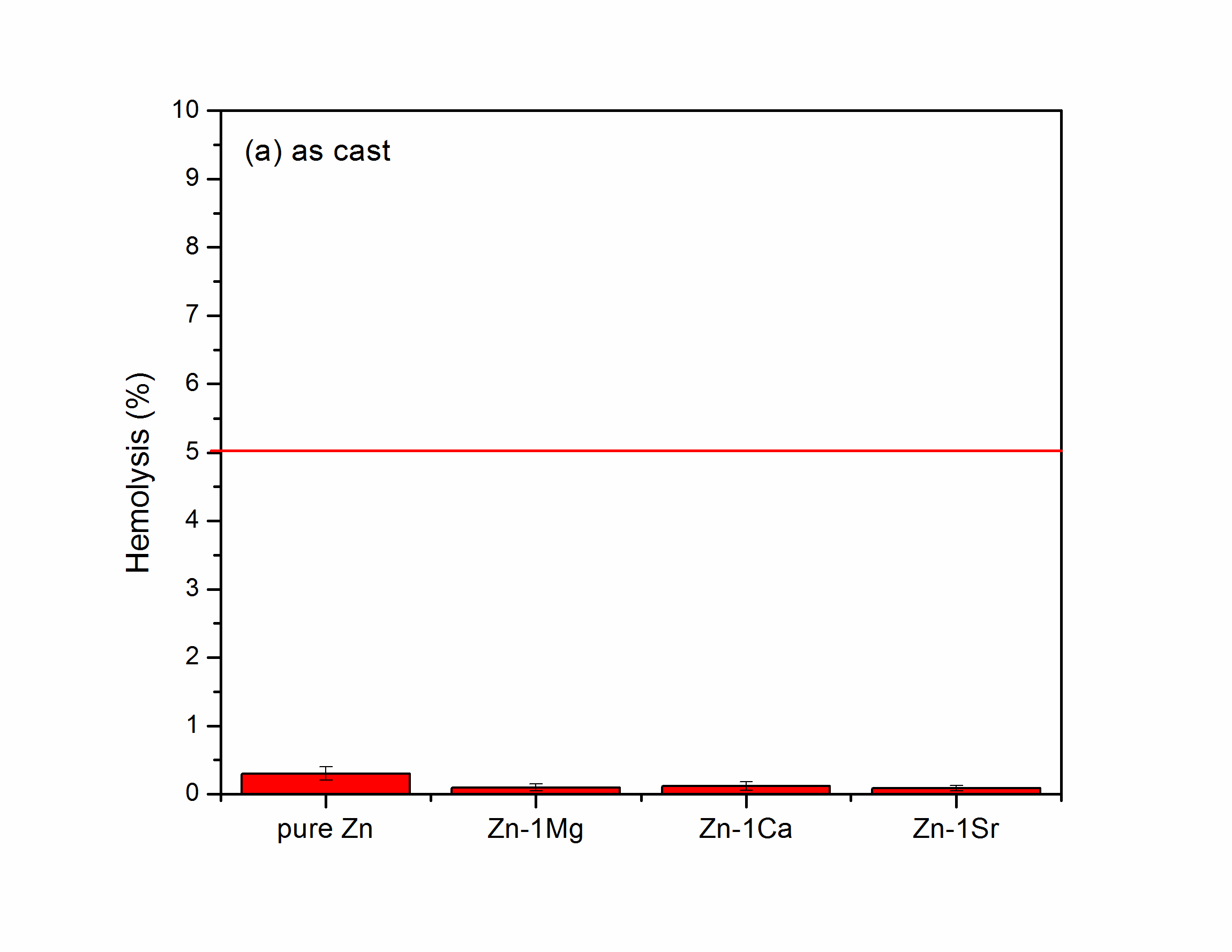

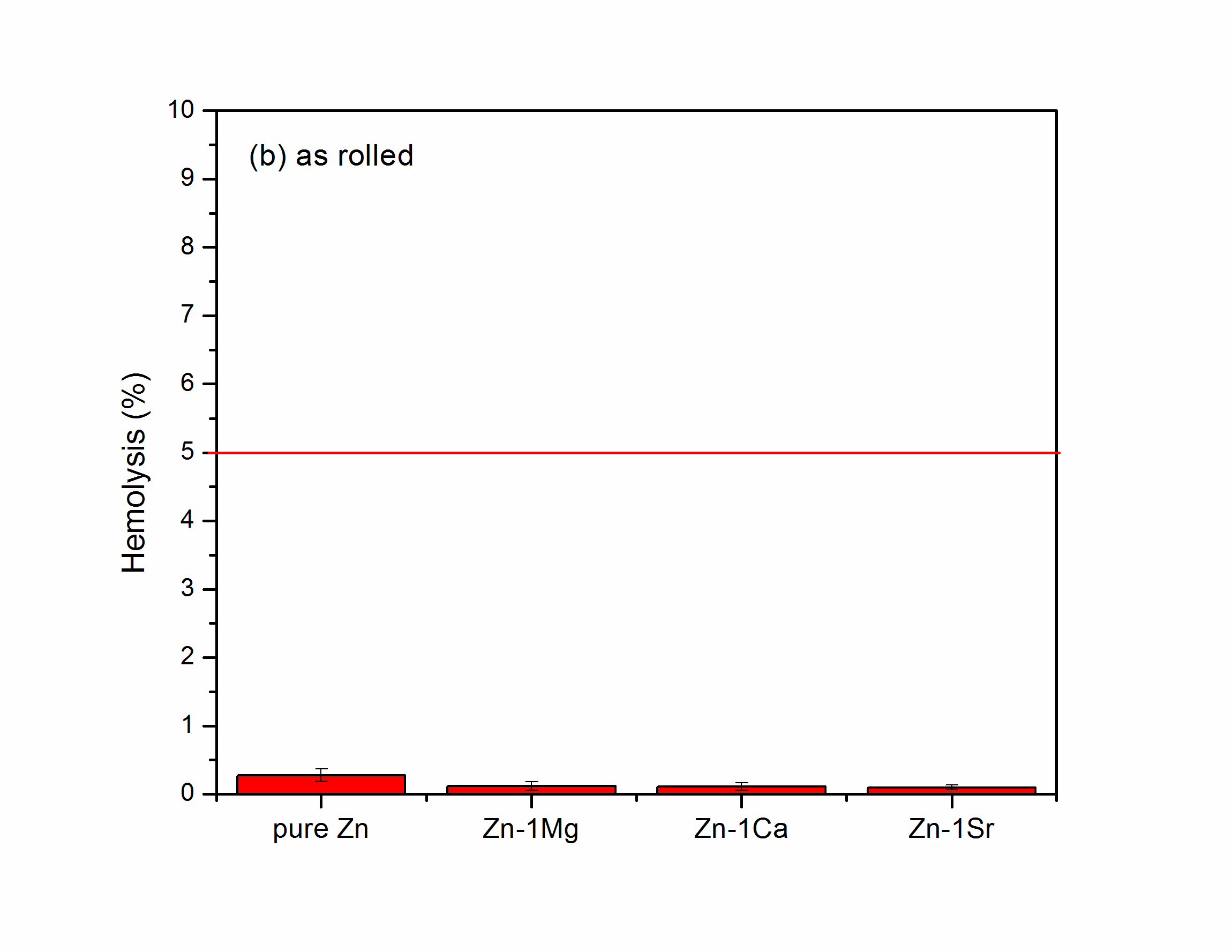


S2 Hemolysis rate of Zn alloys, (a) as-cast, (b) as-rolled.


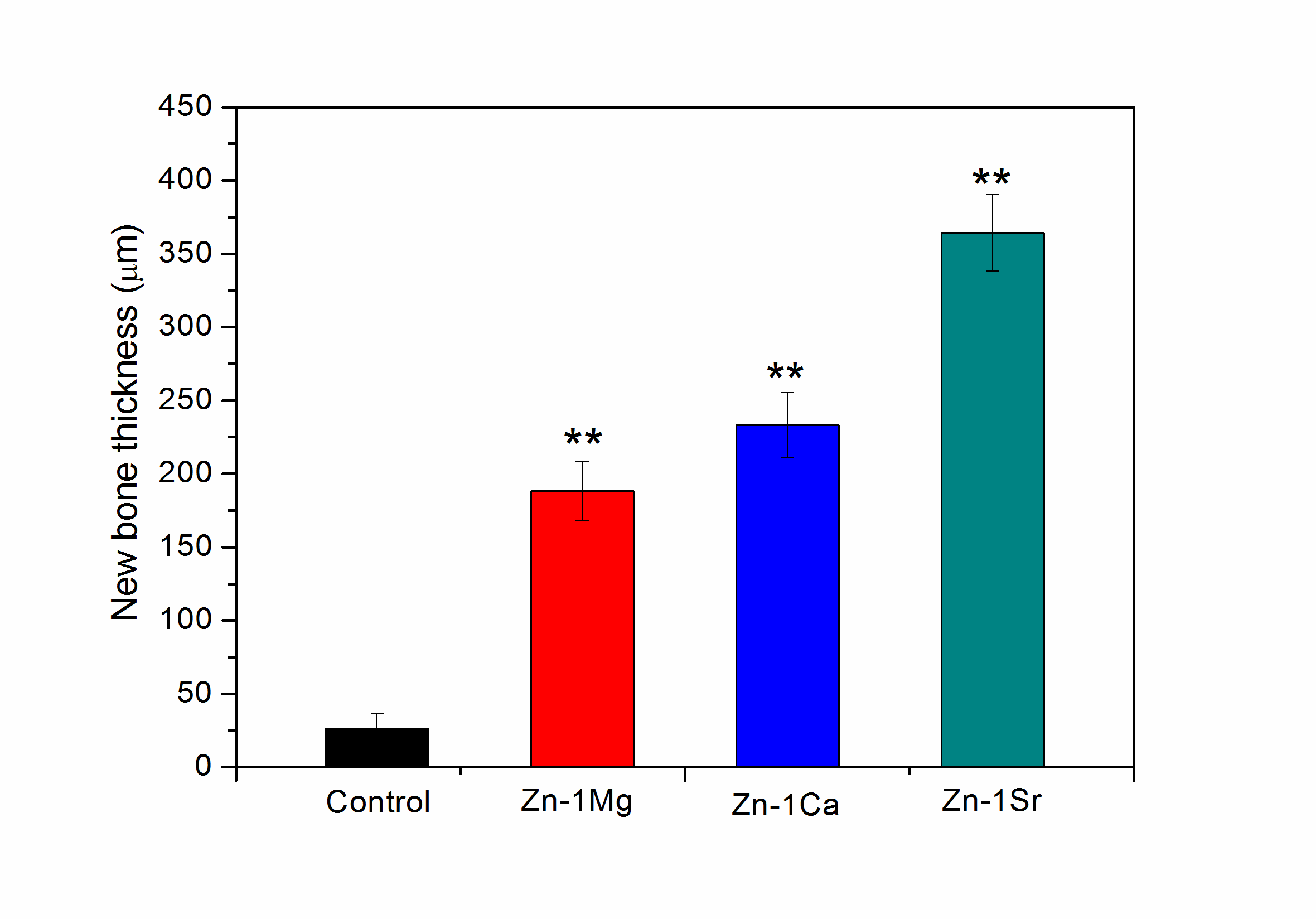


S3 New bone thickness calculated from Fig. 8.
